# Fractional carbon dioxide laser treatment of hypertrophic scar clinical and histopathological evaluation

**DOI:** 10.1007/s10103-025-04371-5

**Published:** 2025-03-12

**Authors:** Zaynab Sayed Keshk, Manal Mohammad Salah, Nevien Ahmed Samy

**Affiliations:** National Institute of Laser Enhanced Science, Egypt Cairo University, Al Giza, Egypt

**Keywords:** Fractional carbon dioxide laser, Hypertrophic scar

## Abstract

Hypertrophic scar could be associated with several complications that interfere with patient daily activities, physical and psychological health and impact patient quality of life. Several therapeutics and maneuvers are used for treatment of hypertrophic scar with variable success and side effects. We aim to evaluate safety and efficacy of fractional carbon dioxide laser on treatment of hypertrophic scar both clinically and histopathologically. Hypertrophic scars in each patient of total thirty patients were subjected to random division with sealed envelope into two parts, part A treated with fractional carbon dioxide laser every month for 5 sessions, and part B lift without treatment for control. Hypertrophic scars in each patient were single or multiple, Single scar was more than 15 cm length. Clinical evaluation was done by two blinded dermatologists, using Vancouver Scar Scale (VSS) and Patient and Observer Scar Assessment Scale (POSAS) for each part before, 3 months and 6 months after treatment. Histopathological evaluation was done for each part before and 3 months after treatment by measuring epidermal thickness, collagen area percent, and elastin area percent. The upper significant clinical and histopathological improvement was shown in carbon dioxide laser treated parts than control parts without significant side effects for fractional carbon dioxide laser treatment. Treatment of hypertrophic scar with fractional carbon dioxide laser is beneficial and generally considered safe, with minimal risk of harm when performed with appropriate laser parameters for male and female patients with skin type III and IV, at different ages and different body regions.

## Introduction

Normotrophic scar is the end result of the process of normal wound healing [1]. Deep trauma, wound infection and increased wound tension increase inflammation, prolong inflammatory phase, provoke over activation of fibroblasts, myofibroblasts and excessive fibrosis during proliferative phase of wound healing process, leading to hypertrophic scar formation [2]. Hypertrophic scar is raised above skin surface, limited by borders of initial trauma, firm in consistency and starts to appear four to eight weeks after trauma [3]. Erythema, elevation and tenderness are the main features of immature hypertrophic scar, with maturation fading of erythema, flattening and cessation of tenderness occur usually within six months after trauma, but the time of maturation can extend more than two years and incomplete flattening can become permanent [4]. Hypertrophic scar Hypertrophic scar could be associated with several complications like contracture, loss of function, disfigurement, pain, itching, longer period of maturation and residual elevated permanent scar [2].Diagnostic tools of hypertrophic scar include invasive biopsy and noninvasive devices like Spectrophotometry for scar color, laser Doppler imaging for scar perfusion, Dermascan for scar thickness and cutometer for scar elasticity [5]. Many therapies are used for hypertrophic scar treatment like corticosteroid injection, 5-Flurouracil, Bleomycin, botulinum toxin, compression therapy, surgical excision cryotherapy, radiotherapy and laser therapy [1, 6]. Fractional carbon dioxide (CO2) 10,600 nm laser is a resurfacing laser that emits pixelated micro beams to deep dermis and absorbed by water, leading to vaporization of micro columns from the scar, coagulation of tissue around vaporized areas and sparing viable tissue columns in between, allowing rapid healing through cellular migration from viable zones to vaporized zones, new collagen and new elastin formation [4], resulted in amelioration of excessive fibrosis of hypertrophic scar and improvement of scar elasticity, thickness, surface irregularities, movement, and enhancement of cosmetic appearance (5, 6, 7).

We aimed to evaluate the efficacy and safety of fractional CO2 laser in treatment of hypertrophic scars in children and adult patients with skin type III and IV both clinically and histopathologically.

### Patient and method

This clinical randomized prospective intra patient controlled study included 30 patients with single or multiple hypertrophic scars of both sex, from different body areas, with Fitzpatrick type III and IV. The study was done in National institute of laser enhanced Science and written informed consent for procedure and photography was taken from guardian of patient below 18 years, and written informed consent was taken for procedure, biopsy and photographing from each patient above 18 years old. The study followed declaration of Helsinki. National institute of laser enhanced science approved the study in February 2020 and the study was from February 2020 to June 2023.

### Inclusion criteria of the scar

The scar duration was less than one year.

Single and multiple, linear and wide hypertrophic scars, the length of single linear scar was more than 15 cm, and the width of single wide scar more than 20 cm2.

### Exclusion criteria

Cutaneous infection, hypersenensitivity reactions, active autoimmune disorders, pregnancy and severe systemic illness.

## Method

Single or multiple Lesions In the same patient were randomly divided with sealed envelope into two parts, part treated with fractional CO2 laser and control part without treatment. Full medical history, history of scar, systemic and cutaneous examinations were done for each patient before treatment.

### Treatment protocol

The session were done every month for five months. The lesion were divided with sealed envelope into two parts (treated part and control part).Topical EMLA cream 5% was applied under occlusion to the treated areas 60 min before procedure, then disinfection of whole lesion with alcohol followed by saline. The treated part for each patient subjected to fractional CO2 10,600 nm laser (D.S.E Seoul, Korea) 40 mJ, density of dots 25/ cm2, depth 3 and size and shape of scanning area differed according to shape and width of the treated part. The control part left without treatment, and covered during treatment. After each treatment, ice cooling for part A and part B, topical antibiotic for five days and sun screen for part A and part B for sun exposed lesions every 2 h were applied.

### Evaluation method

#### Clinical evaluation

Vancouver Scar Scale VSS [8], Patient and Observer Scar Assessment Scale POSAS [9] and photographs were used for clinical evaluation by two blinded dermatologists. Components of VSS evaluated before, 3months and 6 months after treatment.

Components of VSS were vascularity, pigmentation, height, pliability and total score.

Observer components of POSAS were vascularity, pigmentation, thickness, relief, pliability, surface area, total score and observer overall opinion.

Patient components of POSAS evaluated opinion and satisfaction of the patients about scar shape and symptoms, they included pain, itching, color, stiffness, thickness, irregularity, total score and patient overall opinion.

Photographs were taken before treatment and after 6 months from last treatment.

#### Histopathological evaluation

Four mm punch biopsies were obtained from 10 adult patients from each part before, and 3 months after treatment. Formalin fixed, paraffin embedded sections were prepared from each specimen. Sections from each specimen were stained with H&E for epidermal thickness, Masson trichrome stain for collagen, and Orcein stain for elastin. The histopathological examination was done using Leica DM 2500 microscope (x200) and image j analyzer 1.53esoftware USA for detection of collagen area percent and elastin area percent.

### Statistical analysis

Data were revised, coded and tabulated using SPSS software version 20, IBM Corp. Release 2011 (SPSS Statistics for Windows; Armonk, New York, UA).

Repeated measure ANOVA was used to test variables at different time points. Normality hypothesis was tested using Shapiro-Wilk test. Parametric numerical data were experssed with mean ± SD, while nonparametric numerical data were expressed with median (interquartile range).

Paired T-test was used to evaluate statistical significance of differences between means of matched groups. P-value < 0.05 was considered statistically significant, P-value < 0.01 was considered highly significant and p-value > 0.05 was considered insignificant.

### The results

Thirty hypertrophic scar patients from different body regions, females were 56.6% and males were43.3%, with Fitzpatrick type III and IV, their mean age was 16 ± 5 years ranged from 7 to 30 years, the scars were from different body sites, caused by various types of injuries and the mean scar duration was 9 ± 2 months (Table 1).

**Table 1:** Demographic data of the patients

Age	Mean age	16 ± 5 year
Gender	Male	43.3% [13]
	Female	56.6% [17]
Duration of trauma	Mean duration	9 ± 2 months
Cause of injury	Scalds, corrosives and fire	53.3% [16]
	Cutaneous inflammation	3.3% [1]
	Surgical intervention	13.3% [4]
	Lacerations and scratches	29.9% [9]
Scar location	Head and neck	16.66% [5]
	Shoulder	3.3% [1]
	Upper limb	29.69% [9]
	Upper back	3.3% [1]
	Lower abdomen	6.66% [2]
	Buttock	6.66% [2]
	Lower limb	33.3% [10]

#### Clinical evaluation

was done with two blinded dermatologists, using VSS and POSAS before, 3 months and6 months after treatment.

The two groups showed significant improvement in all scar parameters except for pigmentation (*P* > 0.05 for pigmentation parameter three months after last treatment in both VSS, observer component of POSAS and patient component of POSAS (included Overall opinion of the patients) with upper statistical significant improvement in areas treated with fractional CO2 laser than control areas that left without treatment. Six months after last treatment, areas treated with fractional CO2 laser showed upper statistical significant improvement than 3 months after last treatment, while control areas showed no significant improvement between 3 months and 6 months after treatment.

Before treatment VSS was 8 ± 0.36 for both treated areas and control areas, after 3 months from last treatment VSS was 6.53 ± 0.3 for treated area and 7.51 ± 0.4 for control area, and after 6 months from last treatment months VSS was 5.50 ± 0.34 for treated area and 7.51 ± 0.4 for control area (P values < 0.05). Before treatment Patient component of POSAS was 7.5 ± 0.21 for both treated area and control area, after 3 months of last treatment Patient component of POSAS was 6.50 ± 0.21 for treated area, and 6.9 ± 0.31 for control area, and after 6 months Patient component of POSAS was 6.10 ± 0.28 for treated area and 6.9 ± 0.31 for control area (P values < 0.05).

Before treatment Observer component of POSAS was 7.1 ± 0.2 for both treated area and control area, after 3 months from last treatment Observer component of POSAS was 6.13 ± 0.18 for treated area and 6.7 ± 0.19 for control area, and after 6 months from last treatment Observer component of POSAS was 5.70 ± 0.18 for treated area and 6.7 ± 0.19 for control area (P values < 0.05) (Tables 2 and 3).

**Table 2:** Clinical evaluation of control group and fractional CO2 laser treated group before, 3 months and 6 months after treatment

Clinical evaluation		Control areas (*n* = 30)	Fractional CO2 treated areas	p-value
		Mean ± SD	Mean ± SD	
VSS	Before	8 ± 0.36	8 ± 0.36	1
	3 M	7.51 ± 0.4	6.53 ± 0.3	$\:<$0.001**
	6 M	7.51 ± 0.4	5.50 ± 0.34	$\:<$0.001**
	p-value	0.002*	$\:<$0.001**	
POSAS Patients	Before	7.5 ± 0.21	7.5 ± 0.21	1
	3 M	6.9 ± 0.31	6.50 ± 0.21	0.006*
	6 M	6.9 ± 0.31	6.10 ± 0.28	$\:<$0.001**
	p-value	0.005*	$\:<$0.001**	
POSAS Observers	Before	7.1 ± 0.2	7.1 ± 0.2	1
	3 M	6.7 ± 0.19	6.13 ± 0.18	0.001**
	6 M	6.7 ± 0.19	5.70 ± 0.18	$\:<$0.001**
	p-value	0.010*	$\:<$0.001**	

Repeated measure ANOVA * Significant at $\:\mathrm{p}-\mathrm{v}\mathrm{a}\mathrm{l}\mathrm{u}\mathrm{e}<0.05$ ** Significant at $\:\mathrm{p}-\mathrm{v}\mathrm{a}\mathrm{l}\mathrm{u}\mathrm{e}<0.001$

**Table 3:** Histopathological evaluation of control group and fractional CO2 laser treated group before and 3 months after treatment, using H&E for epidermal thickness, Masson trichrome stain and image j analyzer for collagen area percent and orcein stain and image j analyzer for elastin area percent

Histopathological evaluation		Control group (*N* = 10) Mean ± SD	Fractional CO2 laser treated group (*n* = 10) Mean ± SD	*P*-value
Epidermal thickness	Before treatment	139 ± 12	139 ± 12	1.000
	after treatment	124 ± 9	107.95 ± 11.47	< 0.001*
	P-value	< 0.001*	0.001*	
Collagen area percent (using Masson trichrome stain) and image j analyzer	Before treatment	58 ± 6	58 ± 6	1.000
	after treatment	53 ± 7	41.18 ± 2.13	< 0.001*
	P-value	0.003*	< 0.001*	
Elastin area percent (using Orcein stain) and image j analyzer	Before treatment	0.40 ± 0.05	0.40 ± 0.05	1.000
	after treatment	1.40 ± 0.4	2.87 ± 0.4	< 0.001*
	P-value	< 0.001*	< 0.001*	

Paired-sample t-test *P < 0.05 is considered statistically significant

#### Histopathological evaluation

was done before treatment and tree months after treatment for both treated areas and control areas.

Collagen area percent using Masson trichrome stain and image j analyzer, and showed significant improvement in fractional CO2 treated areas and control areas with upper significant improvement in fractional CO2 laser treated areas than areas left without treatment. Before treatment was 58 ± 6 for both treated area and control area, after 3 months from last treatment was 41.18 ± 2.13 for treated area and 53 ± 7 (P values < 0.05).

Elastin area percent using Orcein stainand image j analyzer, and showed significant improvement in fractional CO2 laser treated areas and control areas with upper significant improvement in fractional CO2 treated areas than areas left without treatment. Before treatment was 0.40 ± 0.05 for both treated area and control area, after 3 months from last treatment was 2.87 ± 0.4 for treated area and 1.40 ± 0.4 for control area (P values < 0.05).

**Fig. 1:**
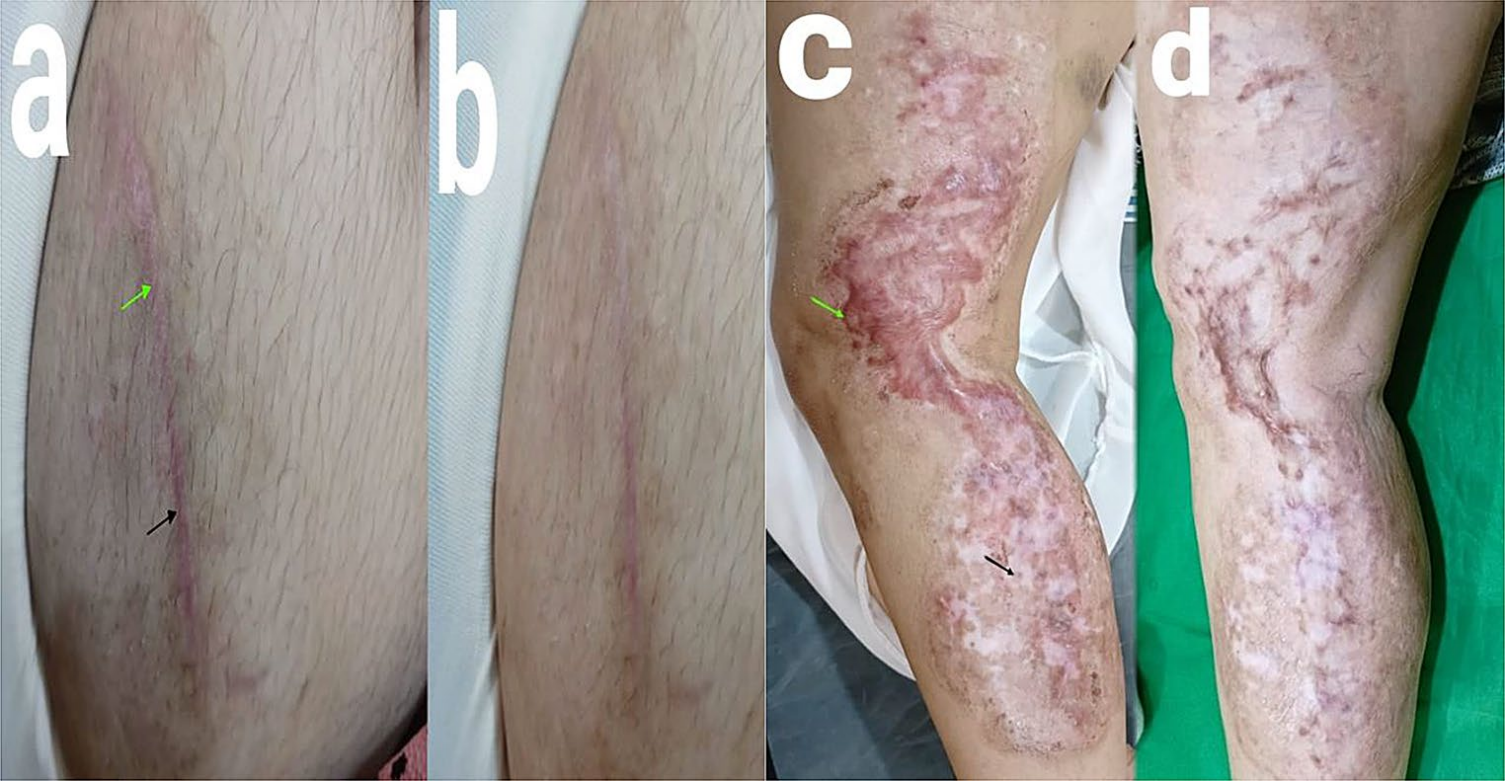
Patient with hypertrophic scar in lower abdomen (**a**) before treatment and (**b**) six months after treatment. Another patient with lower limb hypertrophic scar, (**c**) before treatment and (**d**) six months after treatment. Green arrows refer to parts treated with fractional CO2 laser, while black arrows refer to control parts that showed the spontaneous scar improvement that showed less significant clinical improvement than the laser treated parts

**Fig. 2:**
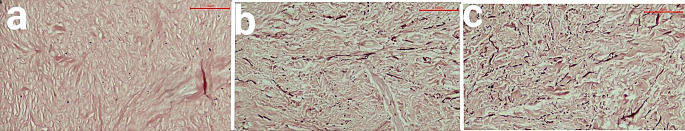
Orcein stained sections (Orcein X20) from the same patient for detection of elastin, (**a**) before treatment, (**b**) from control area 3 months after treatment and (**c**) from fractional CO2 laser treated part 3months after last session and showed more increase in elastin formation than the control part

**Fig. 3:**
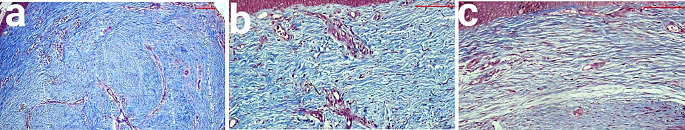
Masson Trichrome stained sections (Masson Trichrome X20) from the same patient for detection of collagen, section (**a**) before treatment and showed collagen nodules, section (**b**) 3 months after treatment from control area and section (**c**) from laser treated area 3 months from last session and showed more parallel collagen fibers

There were no significant differences in improvement between groups as regard age, gender, body region, cause of trauma or duration of hypertrophic scar (P values > 0.05). There was no significant correlation between the severity levels and the results in both fractional CO2 laser treated areas and control areas (P values > 0.05) (See Figs. 1, 2 and 3).

## Discussion

Although spontaneous improvement of hypertrophic scar occurs in many cases, complications from these scars can interfere with patient, s physical, psychological, social and working conditions [10]. The severity, maturation and response to treatment of hypertrophic scar can be assessed clinically, through biopsy and noninvasive objective devices [8, 9]. Several clinical scales are used for hypertrophic scar assessment as VSS which is the most prevalent scale for hypertrophic scar evaluation, and POSAS that included detailed and overall opinion of both patients and physician (8, 9, 11, 12). Biopsy for histopathological examination evaluates changes in collagen and elastin in different stages of the scar, assesses response to treatment and differentiates hypertrophic scar from other pathological conditions (1, 2, 12). Noninvasive objective devices can provide accurate assessment of changes in erythema, thickness and pliability of the scar before and after treatment [5]. Multimodal ultrasound combines traditional ultrasound with other imaging modalities to evaluate thickness, vascularity and elasticity of hypertrophic scar before and after treatment [13, 14]. Disadvantages of multimodal ultrasound include high cost, increased complexity, potential artifacts and patient discomfort due to extended period of examination [14]. Optical coherence tomography (OCT) is a high resolution laser surface imaging that provides accurate measurement of scar thickness and vascularity [15]. Disadvantages of OCT include high cost, limited depth of penetration, limited field of view, artifacts from patient, s movement and potential misdiagnosis [16, 17]. Optical Diffraction tomography (ODT) combines optical coherence tomography with Doppler analysis to provide accurate and immediate measurement of blood flow of hypertrophic scar [18]. Disadvantages of ODT include high cost, limited penetration depth, limited field of view and motion artifacts [18]. Treatment of hypertrophic scar involves surgical excision with high risk of recurrence rate, medical therapies like corticosteroid injection with risk of atrophy, telangiectasia and leukoderma, and silicon based products with risk of allergy and maceration [10, 11]. Bleomycin and 5- fluorouracil are used for hypertrophic scar treatment [1, 12]. Injection of 5- fluorouracil can induce ulceration, diarrhea, leukopenia and neuropathy, and bleomycin can cause infection, anemia, renal and neurological symptoms [1, 12]. Physical maneuvers like compression therapy are used to treat hypertrophic scar with anecdotal effect [19, 20]. Erythema and excessive fibrosis of hypertrophic scar could be treated with different laser devices [1, 6]. Laser targets specific chromo phore without damaging the surrounding tissues [21]. Pulsed dye (PDL) laser, long- pulsed neodymium- dopped yettrium aluminium garnet (Nd: YAG) laser, Erbium YAG laser and f fractional CO2 laser are used for hypertrophic scar treatment [1, 6]. Pulsed dye laser 585 nm and 595 nm absorbed by hemoglobin, leading to coagulation of excess blood vessels and cessation of profibrotic process [10]. Early treatment of hypertrophic scar with Pulsed dye laser ameliorates erythema, fibrosis and accelerates scar maturation [11, 13]. Long- pulsed Nd: YAG laser 1064 nm passes deep in scar tissue, coagulates blood vessels, decreases inflammation and allows collagen remodeling [13].Ablative fractional Erbium: YAG 2940 nm highly absorbed by water, leading to less thermal diffusion, allowing rapid healing and less side effects and induces collagen remodeling [1, 12]. Fractional CO2 laser 10,600 nm resurfaces hypertrophic scar, decreases fibrosis and vascularization, because fractional CO2 laser vaporizes micro columns of the scar and coagulates blood vessels in areas under vaporization [5]. Heat of fractional CO2 laser activates heat shock proteins leading to activation of antifibrotic factors leading to scar renovation through new collagen formation, skin barrier restoration and immune function regulation [6]. Fractional CO2 laser improves Thickness, surface irregularities, skin elasticity, range of motion and cosmetic appearance of hypertrophic scar [1]. No toxicity, no carcinogenicity, no systemic side effects and no harming of areas around scar with fractional CO2 laser (5, 6, 7).The current intra patient controlled randomized study investigated the clinical and histopathological effect of fractional CO2 laser treatment of hypertrophic scars versus the spontaneous modulation of the same scar in the same person. The clinical evaluation in this study was done by using both VSS scale [8], and POSAS scale [9]. Opinion of patients about scar shape, pain and itching and overall opinion were evaluated before, 3 months and 6 months after treatment, and showed more significant improvement in areas treated with fractional CO2 laser 6 months after treatment than the control areas. Vascularity, height, roughness, elasticity, irregularities and thickness of hypertrophic scar were more significantly improved with fractional CO2 laser treated areas, especially 6 months after treatment than control areas, demonstrating the long lasting improvement of hypertrophic scar with fractional CO2 laser. The histopathological examination was done in this study for objective evaluation of the efficacy of fractional CO2 laser treatment of hypertrophic scar versus spontaneous regression of the same scar before and 3 months after treatment, and estimated collagen arrangement, elastin and epidermal thickness changes, using different stains and image j analyzer for precise calculation and analysis, and showed more significant decrease in epidermal thickness, appearance of rete ridges, increase in elastin formation, increase in elastin area percent, improvement of collagen arrangement and decrease in collagen area percent in areas treated with fractional CO2 laser than the control areas. No significant side effects of laser therapy was shown during treatment or follow up in this study. All patients included in this study tolerated fractional CO2 laser treatment and continued follow up without withdrawal, and no recurrence was shown during treatment or follow up period. The higher energy of fractional CO2 laser could induce prolonged pain and erythema, blister formation, prolonged period for recovery and dyspigmentation [7]. Proper selection of patients, proper choice of laser device parameters and following postoperative instructions can avoid laser side effects [21]. The energy of fractional CO2 laser used in this study was 40mj which considered relatively low energy to avoid side effects induced by high fractional CO2 energy [22]. Won et al., treated pediatric patients with hypertrophic scars with low energy fractional CO2 laser, and evaluated scars clinically with VSS and POSAS and they found satisfactory results without side effects [23]. In a comparative study done by Yassen et al., to evaluate fractional CO2 laser versus nanofat on burn scars involving hypertrophic scar, using VSS and POSAS, they found improvement of scar parameters with fractional CO2 laser using POSAS, without significant side effects of laser therapy [24]. Tan et al., in a retrospective study, using both deep and superficial modes of frctional CO2 laser on the same scar, and demonstrated significant effect of fractional CO2 laser on early stage hypertrophic scar with few transient side effects in the form of swelling, seepage and, bleeding [25]. Relatively low energy of fractional CO2 laser was used in the current study and showed no reported cases of seepage, swelling or bleeding. Tawfic et al. showed improvement of hypertrophic scar with fractional CO2 laser in a short term follow up uncontrolled comparative study on keloid and hypertrophic scars [26], The current study showed with both clinical and histopathological assessment of hypertrophic scar before and after treatment with fractional CO2 laser, that fractional CO2 laser is beneficial and generally considered safe for adult and pediatric male and female patients with hypertrophic scars from different body areas with Fitzpatrick type III and IV.

### Limitation of the study

Small sample size and lack of objective noninvasive devices for scar assessment.
